# ER Stress-Perturbed Intracellular Protein O-GlcNAcylation Aggravates Podocyte Injury in Diabetes Nephropathy

**DOI:** 10.3390/ijms242417603

**Published:** 2023-12-18

**Authors:** Shicong Song, Tiantian Hu, Xu Shi, Yongjie Jin, Sirui Liu, Xuehong Li, Wei Zou, Cheng Wang

**Affiliations:** 1 Division of Nephrology, Department of Medicine, The Fifth Affiliated Hospital Sun Yat-Sen University, Internal Medicine Building Room #606, 52 Meihua Dong Road, Zhuhai 519000, China; songshc@mail2.sysu.edu.cn (S.S.); shix37@mail2.sysu.edu.cn (X.S.); jinyj7@mail.sysu.edu.cn (Y.J.);; 2 Guangdong Provincial Key Laboratory of Biomedical Imaging, The Fifth Affiliated Hospital Sun Yat-Sen University, Zhuhai 519000, China

**Keywords:** podocyte injury, diabetes nephropathy, ER stress, O-GlcNAcylation

## Abstract

Diabetes nephropathy (DN) is the leading cause of end-stage renal disease (ESRD) worldwide, and podocyte injury is the central contributor to the progression of DN. Despite the emerging evidence that has established the importance of podocyte endoplasmic reticulum (ER) stress in the pathogenesis of DN, abnormal protein O-GlcNAcylation is also augmented. Currently, the mechanism associating these two hyperglycemia-induced disorders remains poorly understood. This study intended to elucidate whether ER stress drives hyper-protein O-GlcNAcylation to cause podocyte injury in DN. We used both type 1 and type 2 DN models to confirm the occurrence of ER stress and excessive protein O-GlcNAcylation, and then podocyte purification was also conducted for further investigation. Nephroseq V5 data were mined and in vitro studies were applied to reveal the involvement of ER stress and hyper-O-GlcNAcylation in podocyte injury. Our results indicated that ER stress was induced in both type 1 and type 2 DN, and the human RNA-seq data from Nephroseq V5 showed that O-GlcNAcylation-related genes were significantly upregulated in the DN patients. We further demonstrated that ER stress occurred prior to hyper-O-GlcNAc modification and that pharmacologically inhibited protein O-GlcNAcylation can help decrease the podocyte apoptosis induced by hyperglycemia. Together, these discoveries will aid in uncovering the activation of the ER stress–O-GlcNAcylation axis in podocyte injury under DN, which will help open up new therapeutic approaches for preventing DN progression.

## 1. Introductions

Diabetes nephropathy (DN) is the leading cause of end-stage renal disease (ESRD) worldwide, and its incidence has been continuously rising in recent decades [1,2]. Even though the underlying molecular mechanisms predisposing individuals to the onset and progression of DN have been actively investigated, the pathophysiological mechanisms remain incompletely understood. Recently, endoplasmic reticulum (ER) stress was evidenced to play a critical role during the development and progression of DN [3,4], and two compounds, tauroursodeoxycholic acid (TUDCA) and phenylbutyric acid, were found to rescue DN progression by enhancing the ER’s adaptive response [5,6,7], indicating that targeting ER stress may be a promising therapeutic approach to suppress the development of DN.

The ER is an important organelle responsible for protein homeostasis (proteostasis), mediating protein folding, protein modification (for example, glycosylation), protein maturation, and intracellular calcium homeostasis. Among all the biological functions of the ER, protein folding is the well-established process mediated by various folding enzymes and chaperones. Pathophysiological states, such as diabetes, would increase the demand for protein folding or disrupt the normal folding processes, leading to the accumulation of misfolded proteins and, finally, resulting in ER stress [3,8]. As highly specialized and terminally differentiated epithelial cells with high levels of anabolic and catabolic activities [9], podocytes are thought to be highly susceptible to ER stress [7,10,11,12]. Previous studies have shown that enhanced ER stress chaperones (e.g., GRP78 and KDEL) and unfolded protein response (UPR) stress response arms (e.g., ATF6, Xbp1, and eIF2α) were detected in injured podocytes [10,13]. ER stress in podocytes initiates potentially fatal cellular processes such as apoptosis, senescence, and inflammation. Protein O-GlcNAcylation is also increased under ER stress conditions [14,15] because the highly expressed Xbp1 is a direct transcriptional activator of glutamine fructose-6-phosphate aminotransferase 1 (GFAT1), the rate-limiting enzyme of the hexosamine biosynthetic pathway (HBP) upstream of O-GlcNAcylation [16]. Currently, the involvement of ER stress and protein O-GlcNAcylation in podocytes remains largely unclear.

Protein modification by a single O-linked N-acetyglucosamine (O-GlcNAc) moiety at serine or threonine residues, termed O-GlcNAcylation, is one of the post-translational modifications (PTMs) regulating various biological processes including transduction, transcription, and translation [17,18,19]. Acting at serine or threonine residues, as a modification similar to phosphorylation, O-GlcNAcylation is regulated by two enzymes, O-GlcNAc transferase (OGT) and O-GlcNAcase (OGA), whereas hundreds of specific kinases and phosphatases tightly regulate protein phosphorylation. OGT facilitates the addition of protein O-GlcNAcylation by using uridine diphosphate-N-acetylglucosamine (UDP-GlcNAc) derived from the HBP pathway or, contrarily, the OGA mediates the removal of O-GlcNAcylation from proteins. O-GlcNAcylation senses nutrient states, cellular stress, and immunity responses, and the perturbation of intracellular protein O-GlcNAcylation is highly associated with diabetes, cardiovascular disease, and neurodegeneration [18,20,21,22]. It is well-documented that O-GlcNAcylation levels are increased in experimental diabetes and tissues derived from patients with diabetic kidney disease [23,24], but how hyperglycemia augments protein O-GlcNAcylation remains poorly known.

In this study, using both type 1 and type 2 DN models as well as an analysis of human RNA-seq data from the Nephroseq V5 database, we found that hyper-O-GlcNAcylation was characterized by and mechanistically linked to ER stress in podocyte injury induced by DN. Specifically, we found that hyperglycemia-induced ER stress drove hyper-O-GlcNAcylation, resulting in podocyte injury. Collectively, the current study indicated that targeting protein O-GlcNAcylation in podocytes may be a novel stratagem for preventing DN progression.

## 2. Results

### 2.1. ER Stress Is Induced in Both Type 1 and Type 2 DN

**Figure 1:**
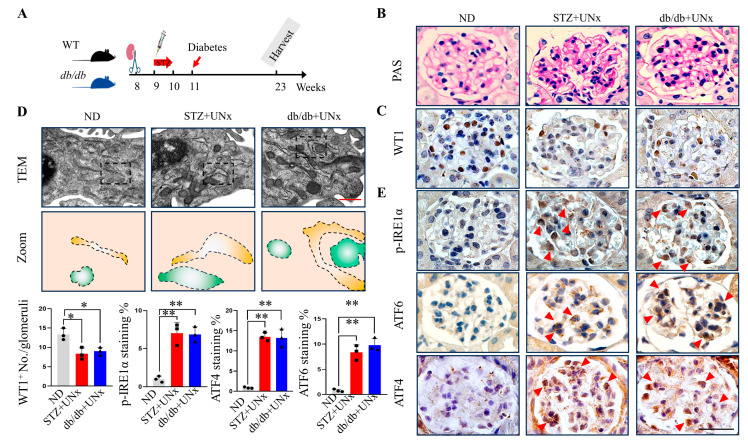
ER stress was induced in both the type 1 and type 2 DN subjects. (**A**) A schematic diagram showing the procedure for inducing the STZ plus UNx and db/db plus UNx DN mice. (**B**) Morphological examinations of the glomerular changes using periodic acid–Schiff (PAS) staining. (**C**) Representative immunohistochemistry images of WT1 in the kidneys from the different groups. The corresponding bar graph is presented in the bottom left of the figure (10 glomeruli per mouse were analyzed, n = 3 mice per group). (**D**) Representative TEM images of the podocytes in the kidneys from the different groups, squares highlight the magnified areas and tracing of the mitochondria and ER from the aforementioned TEM images were shown in lower panel (three podocytes per mouse were analyzed, n = three mice per group). (**E**) Representative immunohistochemistry images of p-IRE1α, ATF6, and ATF4 in the kidneys from the different groups (10 glomeruli per mouse were analyzed, n = 3 mice per group), and the corresponding bar graph is presented in the bottom of the figure. The arrowhead indicates the positive DAB labeling. *, *p* < 0.05 and **, *p* < 0.01 by one-way ANOVA, followed by Tukey tests. The scale bars = 20 µm in (**B**,**C**,**E**) and 0.5 µm in (**D**).

To determine whether ER stress was induced in DN, we employed both type 1 and type 2 diabetes mouse models of DN. As the C57BL/6 mouse is relatively resistant to kidney injury induced by diabetes [25], we applied both multiple low doses of STZ injection and unilateral nephrectomy (UNx) to the C57BL/6 mice to induce type 1 DN [26,27]. Type 2 diabetes was induced using db/db mice with a C57BLKS/J background, and the UNx was performed at the age of 6~8 weeks to induce type 2 DN. DN is defined as a diabetes state maintained for 12 weeks. The animal experimental scheme is presented in Figure 1A. Typical DN lesions were observed in periodic acid–Schiff (PAS) staining, including thickening of capillary basement membranes and glomerular shrinkage (Figure 1B and Figure S1A). The decreased podocytes were confirmed by WT1 immunohistochemistry staining, indicating significant podocyte injuries in the DN mice compared with the non-diabetes (ND) mice (Figure 1C and Figure S1B). The body weights, glucose levels, and kidney function indexes are included in Table 1. A chronic hyperglycemia state is certainly a common feature in type 1 and type 2 diabetes, but the body weights and biochemical characteristics of these two models may have slight differences (Table 1). These differences cannot be explained by hyperglycemia toxicity as there are many mechanisms independent from hyperglycemia involved in the pathogenesis of diabetes [28,29]. We sought to determine the morphological change due to ER stress in the podocytes of the DN mice [12], and the typical ER dilatations were revealed in both the STZ plus UNx and db/db plus UNx groups (Figure 1D and Figure S1C). The following three signaling pathways were activated when ER stress was initiated: the IRE1α–XBP1 pathway, the ATF6 pathway, and the PRKR-like ER kinase (PERK)–eIF2α–ATF4 pathway [12,30]. Our immunohistochemistry staining results indicated that phosphorylated IRE1α (p-IRE1α) was increased in both the STZ plus UNx and db/db plus UNx groups (Figure 1E, upper panel, and Figure S2A), which was consistent with a previous study where the IRE1α–XBP1 pathway was activated in DN subjects [3]. We tested the other two ER stress pathways using ATF6 and ATF4 antibody labeling, respectively. Consistent with others’ findings, both ATF6 and ATF4 were upregulated in both the STZ plus UNx and db/db plus UNx groups (Figure 1E, middle and bottom panels, and Figure S2B,C) [12]. Collectively, these findings indicated that ER stress had been induced in both the type 1 and type 2 DN subjects.

### 2.2. Increased Protein O-GlcNAcylation Is Identified in the Glomeruli of the DN Mice

**Figure 2:**
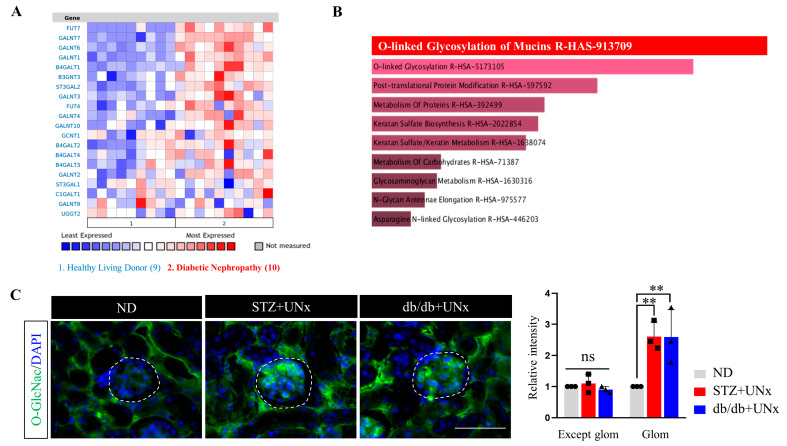
Increased protein O-GlcNAcyaltion was identified in the glomeruli of the DN mice. (**A**) RNA-seq data from Nephroseq V5 compared to the expression profiles of the protein O-GlcNAcylation-related genes of the healthy kidney donors and the DN patients. (**B**) Pathway enrichment of the different expression genes from Nephroseq V5. (**C**) Representative immunofluorescence images of O-GlcNAc staining and the quantification of the fluorescence intensity in the kidneys from the different groups, dash circle highlight the glomerulus. (we randomly selected five fields of view per mouse for analysis, n = 3 mice per group). Glom, glomerulus. ns > 0.05, ** *p* < 0.01 using one-way ANOVA, followed by Tukey tests. The scale bar = 20 µm.

It was recently reported that the increased expression of Xbp1 under an ER stress condition would activate the HBP pathway by binding to the promoter of GFAT1 [16]. As a speed-limited enzyme of the HBP pathway, GFAT1 mediates the production of UDP-GlcNAc, the donor substrate of protein O-GlcNAcylation. Therefore, ER stress would be correlated with increased O-GlcNAcylation modification. To test the O-GlcNAcylation activity in humans, we used Nephroseq V5 to compare the expression profiles of protein O-GlcNAcylation-related genes between healthy kidney donors and DN patients. The available RNA-seq data in Nephroseq V5 indicated a higher expression level of the O-GlcNAcylation gene in the DN patients (Figure 2A), all of which enriched the protein O-GlcNAcylation pathway (Figure 2B). To further confirm the expression pattern of O-GlcNAc in animal models, we conducted immunofluorescence measurements. Surprisingly, no significant fluorescence differences were detected in the tissues other than in the glomeruli between the ND and DN groups (Figure 2C and Figure S3A). Due to the high energy demands of renal tubulars, we thought the explanation was that the increased glucose uptake was maintained in the renal tubular structures, resulting in increased glucose fractions entering the HBP pathways, leading to enhanced O-GlcNAcylation modification under a normal condition. Under diabetes, the glucose absorption and/or OGT modification capacity was saturated [31], which implied that it was nearly impossible to further improve the modification ability. Consequently, no significant differences were observed between the ND and DN groups. However, increased O-GlcNAcylation was detected in the glomeruli of both the STZ plus UNx and db/db plus UNx groups (Figure 2C, highlighted by the dashed circle), implying the critical role of O-GlcNAcylation in glomerular pathological changes induced by DN. Collectively, these results revealed that protein O-GlcNAcylation was increased in the DN subjects, especially in their glomeruli.

### 2.3. O-GlcNAc Protein Modification Is Increased in Podocytes

**Figure 3:**
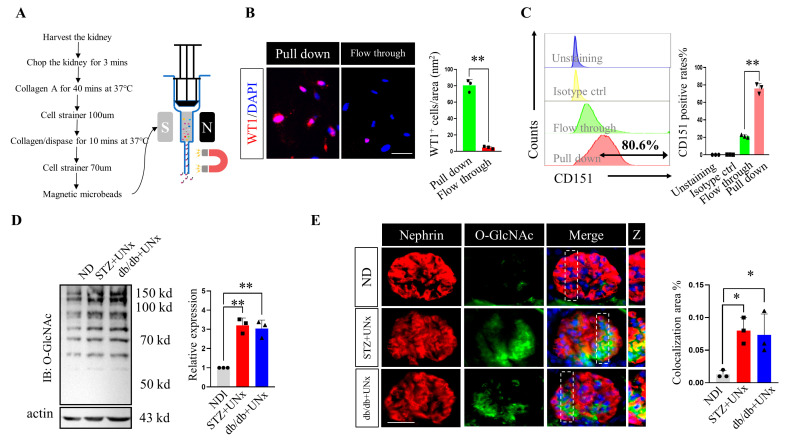
O-GlcNAc protein modification was increased in the subjects’ podocytes. (**A**) A schematic diagram showing the podocyte purification procedure. (**B**) Representative immunofluorescence images of the WT1 staining and the quantification of the positive rates in the pulled-down and flow-through cells (we randomly selected five fields of view per mouse for analysis, n = 3 mice per group). (**C**) Flow cytometry analysis of the CD151 positive cells and the quantification of the positive rates in the different groups (n = 3). (**D**) Representative immunoblotting of O-GlcNAcylation in the pulled-down podocytes and quantification of the expression levels in the different groups (n = 3 blots in total). (**E**) Representative dual-immunofluorescence staining image and quantification of the colocalization areas in the different groups, squares highlight the representative colocalization areas (we randomly selected five fields of view per mouse for analysis, n = 3 mice per group). Z, zoom. * *p* < 0.05 and ** *p* < 0.01 using one-way ANOVA, followed by Tukey tests (**D**,**E**) or Student’s *t*-tests (**B**,**C**). The scale bars = 20 µm.

We showed that protein O-GlcNAc acylation was increased in the DN subjects’ glomeruli, and we sought to clarify whether O-GlcNAc glycation was also increased in their podocytes. CD151 was used as a cell surface marker [32,33] to purify the podocytes using a MACS magnetic column, and the workflow is presented in Figure 3A. Then, we examined the pull-down efficiency using both immunofluorescence and flow cytometry. The immunofluorescence staining showed that the WT1^+^ rate of the CD151 pull-down group was approximately 80% and the flow-through positive rate was approximately 5% (Figure 3B). Following this, the flow cytometry revealed the same results, showing an 80.6% positive rate in the pull-down cells (Figure 3C) and indicating a robust separation efficiency. The immunoblotting using the pulled-down podocytes showed significantly higher global protein O-GlcNAcylation levels in the STZ plus UNx and db/db plus UNx groups (Figure 3D). Further, the dual-immunofluorescence using O-GlcNAc and nephrin showed increased colocalization areas in both the STZ plus UNx and db/db plus UNx groups (Figure 3E and Figure S3B), revealing blooming O-GlcNAcylation in the podocytes. However, we noticed that the nephrin did not perfectly overlap with the O-GlcNAc. There are several reasons for this phenomenon. Firstly, podocytes are not the only cells that undergo O-GlcNAcylation; mesangial cells [34] and/or endothelial cells [23] may also undergo O-GlcNAcylation in the glomeruli. Second, the special morphology of a podocyte, especially its zipper-like foot process structure, makes dual-staining nearly impossible for presenting a complete overlap. Because of these data, we concluded that the protein O-GlcNAc modification was increased in the podocytes.

### 2.4. An Increase in O-GlcNAc Modification Is Correlated with ER Stress

**Figure 4:**
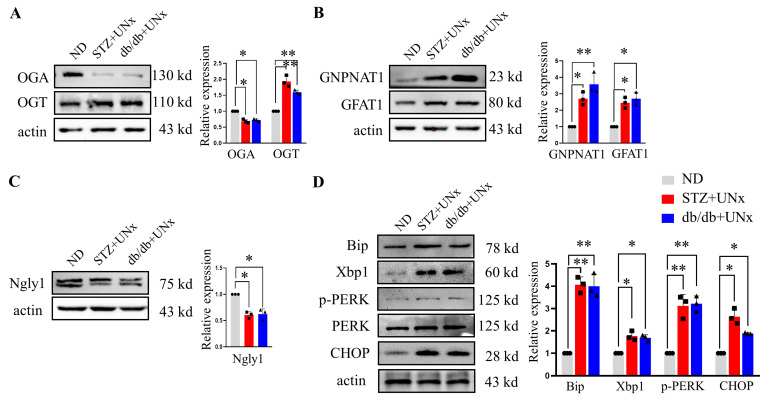
Increased O-GlcNAc modification levels were correlated with ER stress. (**A**) Representative immunoblotting of OGA and OGT and the quantification of the expression levels in the different groups (n = 3 blots in total). (**B**) Representative immunoblotting of GNPNAT1 and GFAT1 and the quantification of the expression levels in the different groups (n = 3 blots in total). (**C**) Representative immunoblotting of Ngly1 and the quantification of the expression levels in the different groups (n = 3 blots in total). (**D**) Representative immunoblotting of Bip, Xbp1, p-PERK, PERK, and CHOP and the quantification of the expression levels in the different groups (n = 3 blots in total). * *p* < 0.05 and ** *p* < 0.01 using one-way ANOVA, followed by Tukey tests.

To test the protein O-GlcNAcylation levels, we utilized the pulled-down podocytes from the different groups for immunoblotting. We first detected the expression levels of OGA and OGT, two critical enzymes that participate in the protein O-GlcNAcylation described above. Significant decreases in OGA expression were observed in the DN group compared with the ND group; furthermore, higher expression levels of OGT were detected in the DN group (Figure 4A). The catalytic activity of OGT is highly sensitive to elevated UDP-GlcNAc levels [35], which is the terminal product of the HBP pathway, and as such, we sought to measure the abundance of two HBP speed-limited enzymes in the pulled-down podocytes. In the podocytes pulled down from the STZ plus UNx and db/db plus UNx subjects’ kidneys, glucosamine-phosphate N-acetyltransferase (GNPNAT1), another key enzyme of the HBP pathway, and GFAT1 were upregulated compared with the pulled-down podocytes from the ND subjects’ kidneys (Figure 4B). These data provided strong evidence for the notion that protein O-GlcNAcylation and HBP are activated in the podocytes of DN subjects’ kidneys. Ngly1 is a highly conserved deglycosylating enzyme that cleaves N-glycans from misfolded glycoproteins to alleviate ER stress [36,37]. We found that the expression of Ngly1 decreased significantly in the podocytes pulled down from the STZ plus UNx and db/db plus UNx subjects’ kidneys compared with the ND subjects’ kidneys (Figure 4C). Moreover, ER stress-related proteins, including Bip, Xbp1, p-PERK, and the C/EBP homologous protein (CHOP), were increased significantly in the DN groups (Figure 4D). These data were consistent with our previous immunohistochemistry staining, which implied that three of the ER stress pathways were activated in the DN subjects. Taken together, we concluded that increased O-GlcNAc modification levels were correlated with ER stress in the podocytes under the diabetes condition.

### 2.5. Pharmacological Mediation of Protein O-GlcNAcylation in Podocyte Injuries

**Figure 5:**
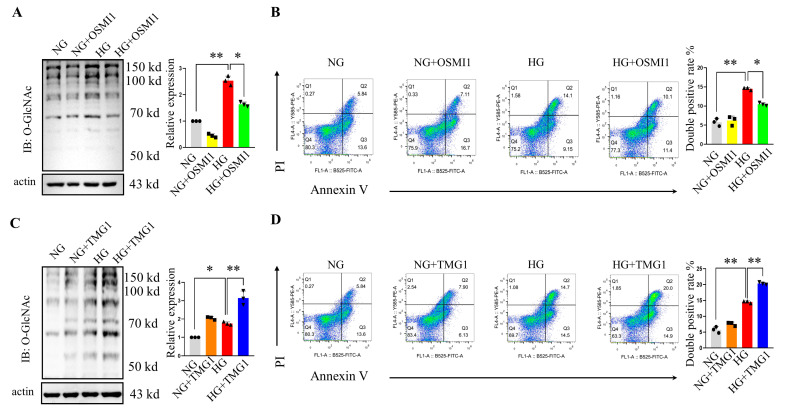
The pharmacological inhibition/activation of protein O-GlcNAcylation alleviated/aggravated podocyte injuries, respectively. (**A**) Representative immunoblotting of O-GlcNAc and the quantification of the expression levels in the different groups (n = 3 blots in total). (**B**) Flow cytometry analysis of the annexin V- and PI-positive cells and the quantification of the double-positive rates in the different groups (n = 3). (**C**) Representative immunoblotting of O-GlcNAc and the quantification of the expression levels in the different groups (n = 3 blots in total). (**D**) Flow cytometry analysis of the annexin V- and PI-positive cells and the quantification of the double-positive rates in the different groups (n = 3). NG, normal glucose; HG, high glucose; TMG, Thiamet-G; PI, propidium iodide. * *p* < 0.05 and ** *p* < 0.01 using one-way ANOVA, followed by Tukey tests.

We next sought to investigate whether pharmacologically mediated protein O-GlcNAcylation is a potential therapeutic approach in podocyte injury induced by hyperglycemia in vitro. Considering that there was a significant increase in OGT expression upon DN exposure, we used OSMI1, a potent protein O-GlcNAcylation inhibitor targeting OGT, to treat the podocytes in a high-glucose medium. Treatment with OSMI1 reduced the abnormally increased O-GlcNAcylation levels efficiently (Figure 5A) in the high-glucose (HG) plus OSMI1 cells compared with the HG cells, and the flow cytometry analysis showed that the inhibition of abnormal protein O-GlcNAcylation using OSMI1 could attenuate the apoptosis induced by hyperglycemia (Figure 5B). Thiamet-G (TMG) is a chemical compound that increases protein O-GlcNAcylation by blocking OGA. The immunoblotting results indicated that treatment with OGA increased O-GlcNAcylation levels efficiently (Figure 5C) in the HG plus TMG group compared with the HG group, and the apoptosis rates induced by the high glucose were obviously aggravated in the HG plus TMG groups (Figure 5D). Collectively, these results suggested that the overactivation of O-GlcNAc modification would aggravate podocyte injuries induced by high glucose, while targeting protein O-GlcNAcyaltion may be a promising therapeutic approach for rescuing DN progression.

### 2.6. ER Stress Drives O-GlcNAc Protein Modification In Vitro

**Figure 6:**
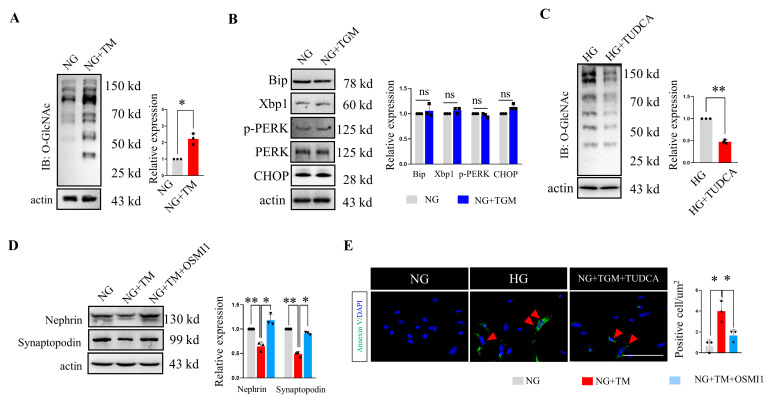
ER stress drove O-GlcNAc protein modification in vitro. (**A**) Representative immunoblotting of O-GlcNAc and the quantification of the expression levels in the different groups (n = 3 blots in total). (**B**) Representative immunoblotting of Bip, Xbp1, p-PERK, PERK, and CHOP and the quantification of the expression levels in the different groups (n = 3 blots in total). (**C**) Representative immunoblotting of O-GlcNAc and the quantification of the expression levels in the different groups (n = 3 blots in total). (**D**) Representative immunoblotting of nephrin and synaptopodin and the quantification of the expression levels in the different groups (n = 3 blots in total). (**E**) Representative immunofluorescence images of the annexin V staining and the quantification of the positive rates from the different groups, arrowheads indicated the positive cells (we randomly selected five fields for analysis, n = 3 mice per group). NG, normal glucose; HG, high glucose; TMG, Thiamet-G; TM, tunicamycin; TUDCA, tauroursodeoxycholic acid. ns > 0.05, * *p* < 0.05, and ** *p* < 0.01 using one-way ANOVA, followed by Tukey tests (**D**,**E**) or Student’s *t*-tests (**A**–**C**). The scale bars = 20 µm.

Having demonstrated that both the ER stress and O-GlcNAcylation were increased under the diabetes condition, we next tested for a link between these two processes. We first treated the podocytes in a normal medium with tunicamycin (TM), a well-established inducer of ER stress, to explore the occurrence of ER stress prior to protein O-GlcNAcylation. The robust upregulation of global O-GlcNAc modification was observed in the NG plus TM cells (Figure 6A), which suggested that the induction of ER stress, whether elicited by high glucose or TM, was correlated with the augmentation of O-GlcNAc modification in the podocytes. However, the upregulation of O-GlcNAcylation with TMG1 in the normal glucose (NG) medium did not show an ER stress signature; specifically, no significant changes in the ER stress markers, including Bip, Xbp1, p-PERK, and CHOP, were observed between the NG and NG plus TMG1 groups (Figure 6B). Contrarily, the high-glucose cultured cells treated with TUDCA, a potent ER stress inhibitor, showed decreased O-GlcNAcyation levels compared with the HG group (Figure 6C). These data suggested that ER stress was, itself, a bona fide trigger of cellular O-GlcNAc protein modification. To further evaluate if the ER stress drove the protein O-GlcNAcylation that resulted in podocyte injury, we detected the nephrin and synaptopodin expression levels using immunoblotting. We found that both the nephrin and synaptopodin expression levels decreased significantly in the podocytes treated with TM, while additional treatment with OSMI1 could alleviate the podocyte injuries reflected by the higher expression levels of both nephrin and synaptopodin (Figure 6D). Furthermore, the annexin V staining showed fewer apoptosis cells in the NG plus TM plus OSMI1 group compared with the NG plus TM group (Figure 6E). These data indicated that the inhibition of protein O-GlcNAcylation by OSMI1 could rescue the podocyte injuries induced by ER stress. Collectively, the ER stress occurred prior to O-GlcNAc modification under the hyperglycemia condition, and ER stress drove the O-GlcNAcylation that resulted in podocyte injuries.

## 3. Discussion

Even though there are compelling past studies that confirm podocyte injury as a critical mediator in the pathogenesis of DN, therapeutic strategies for preventing DN progression by targeting podocyte injury are not currently available. As studies highlighting the link between ER stress and podocyte injury continue to increase, new therapeutic approaches for DN patients will open up. In this study, we showed that podocyte ER stress (morphological and functional) was induced under a DN condition. Further, based on the RNA-seq data from Nephroseq V5 showing higher expression levels of O-GlcNAcylation genes in DN patients, we conducted immunofluorescence staining to confirm the O-GlcNAcylation pattern in the animal model, and we found that greater O-GlcNAc modification occurred in the glomeruli of the DN mice. The podocyte isolation experiments further confirmed the increased protein O-GlcNAcylation in the DN mice, and the elevated O-GlcNAcylation levels were correlated with ER stress. Our study found that ER stress occurred prior to protein O-GlcNAcylation under a high-glucose condition, and pharmacologically inhibiting protein O-GlcNAcylation using OSMI1 could rescue podocyte injury induced by tunicamycin. Collectively, this study established that ER stress drives excessive O-GlcNAcylation to trigger podocyte injury, resulting in DN progression.

In a diabetes condition, glucose will enter podocytes to enhance glycolysis as well as multiple glucose metabolic pathways. HBP, a side branch pathway of glycolysis, utilizes a small fraction (2~5%) of fructose-6-phosphate to produce UDP-GlcNAc, the donor substrate for protein O-GlcNAcylation. The catalytic activity of OGT is highly sensitive to the UDP-GlcNAc level, and as such, O-GlcNAcylation is elevated in response to diabetes [24,35]. Following the above, it is worth noting that it is unclear whether excessive O-GlcNAcylation causes diabetes or is merely an effect of the overall dysfunction caused by diabetes [38]. Our data showed that podocytes with increased global O-GlcNAcylation by the TMG inhibition of OGA under a normal glucose medium did not lead to a higher apoptosis rate compared with the NG group (Figure 5C,D). Further, no significant changes in ER stress proteins were detected in the podocytes treated with TMG1 (Figure 6B). Our results implied that the hyper-O-GlcNAcylation, which was accompanied by hyperglycemia, induced podocyte apoptosis, which, at least in part, resulted from the ER stress. The overactivation of O-GlcNAcylation by TMG1 in the absence of ER stress was not sufficient to cause podocyte apoptosis. Similar findings were reported by Macauley et al., who argued that the treatment of rats and mice with NButGT, another OGA inhibitor, increased global O-GlcNAc levels in multiple organs, but this did not translate to perturbations in the activation of the insulin-signaling pathway, glucose homeostasis, b-cell toxicity, or insulin resistance [39]. Thus, the data from our study and others suggest that O-GlcNAc may be a downstream effect of diabetes and diabetes complications, and hyper-O-GlcNAcylation alone is not sufficient for triggering podocyte injury.

In the present study, we found that ER stress occurred prior to protein O-GlcNAcylation. Both hyperglycemia-induced and tunicamycin-induced ER stress resulted in the augmentation of O-GlcNAc modification in podocytes, while the O-GlcNAcylation inducer TMG alone did not elicit ER stress. These data suggested that the protein O-GlcNAc modification was mediated by ER stress. Similar findings have been reported for elevated O-GlcNAcyaltion in various stress conditions. Zhao V et al. found that Xbp1, the most conserved signal transducer of ER stress, triggered HBP activation and excessive O-GlcNAc modification by mediating the transcription of the rate-limiting enzyme GFAT1. They further established that the ER stress, HBP, and O-GlcNAc modification axis was activated in a variety of stress conditions [16]. Despite the activation of Xbp1, the other two ER stress pathways were also activated under the DN condition [4,13]. A new study using human RNA sequencing data revealed that GNPNAT1, another rate-limiting enzyme of the HBP pathway, was the target gene of PERK [40]. All of these reports have indicated that the pro-apoptotic response caused by ER stress is closely associated with excessive O-GlcNAc modification, but further studies are needed to uncover the downstream mechanism of how O-GlcNAcylation leads to apoptosis.

Hyperglycemia-induced hyper-O-GlcNAcylation is well-documented in DN above and beyond podocytes. Mesangial expansion and hypertrophy are two of the earliest renal abnormalities observed in DN. Masson et al. reported that HBP activation via glucosamine treatment led to mesangial cell cycle arrest at the G0/G1 phase, resulting in the reduced proliferation and induction of hypertrophy [34]. Therefore, these data indicate that excessive protein O-GlcNAcylation is an important contributor to mesangium expansion. In podocytes, Akimoto et al. found that the abnormal O-GlcNAcylation of α-actinin 4 and actin may cause morphological changes in cell shapes and adhesion in the podocyte foot processes, leading to the progression of DN [41]. Na et al. reported that the knockdown of OGT significantly rescued nephrin expression in nephrocytes, cells that are analogous to mammalian podocytes, and it improved the lifespans of Drosophila fed with a high-sucrose diet [42]. Our data also showed that the inhibition of protein O-GlcNAc modification using OSMI1 could efficiently alleviate the podocyte apoptosis induced by high-glucose or tunicamycin treatment, indicating that hyper-O-GlcNAcylation is the executor of DN progression. Collectively, targeting protein O-GlcNAcylation was shown to have a therapeutic effect on glucose toxicity in a hyperglycemia environment.

In summary, this study revealed that hyperglycemia induced ER stress by activating all three conserved signaling pathways, leading to global protein hyper-O-GlcNAcylation and initiating podocyte apoptosis. Both an ER stress inhibitor and an O-GlcNAc modification inhibitor can alleviate podocyte injury in vitro, and this will help deepen our understanding of the mechanisms mediating podocyte injury under a DN condition and lead to novel therapeutics. Further studies are needed to confirm this finding in vivo and explore the underlying mechanism by which O-GlcNAc acylation mediates podocyte injury. Finally, before a highly specific medication becomes available, reducing modifiable ER stress and O-GlcNAcylation exposures may be an effective approach for improving clinical outcomes in DN.

## 4. Materials and Methods

### 4.1. Animal Study

All the animal experiments were approved by the Institutional Animal Care and Use Committee of the Sun Yat-Sen University School of Medicine (Document #00327). The animal procedures were performed in accordance with the National Institutes of Health (NIH) Guide for the Care and Use of Laboratory Animals. All the mice were housed on sawdust bedding in a pathogen-free facility (12 h light/dark cycle) with free access to water and a chow diet. For all of the in vivo experiments, 6~8-week-old male mice were used. The number of mice used for the experiments is indicated in the figure legends.

### 4.2. STZ Plus Unilateral Nephrectomy-Induced Diabetic Kidney Nephropathy Model

C57BL/6 mice are relatively resistant to diabetic nephropathy induced by STZ [25], so we set up the type 1 diabetes (T1D)-induced nephropathy by subjecting the C57BL/6 mice to both low-dose STZ and unilateral nephrectomies (UNx) [25,26,27] (the mice were purchased from Yangcheng Biotechnology, Guangzhou, China). Six-to-eight-week-old male mice were subjected to UNx and allowed to recover for one week. They were continuously intraperitoneally injected with low-dose STZ (50 mg/kg, Cat#18883-66-4, Sigma, St. Louis, MO, USA) 5 times to induce partial insulin deficiency (or they were injected with the control vehicle). Type 1 DN was defined as a diabetes state maintained for 12 weeks. At the end of the study, the kidneys were dissected for further analysis.

### 4.3. Unilateral Nephrectomy-Induced Diabetic Kidney Nephropathy in db/db Mice

The db/db mice with a C57BLKS/J background (purchased from Yaokang Biotechnology, Guangzhou, China) were used for the type 2 DN model. Briefly, unilateral nephrectomies were conducted on db/db male mice at 6~8 weeks of age, and then the db/db mice were maintained for another 12 weeks when the type 2 diabetic kidney nephropathy was defined. At the end of the study, the kidneys were dissected for further analysis.

### 4.4. Immunofluorescence Staining

The formalin-fixed, paraffin-embedded (FFPE) sections were deparaffinized and rehydrated as previously described [43,44]. Antigen retrieval was performed by autoclaving at 120 °C for 15 min in citrate buffer, followed by 0.2% Triton X-100 permeabilization for 10 min. The slices were incubated with blocking solution containing 10% donkey serum for 1 h, and then they were combined with the primary antibodies (O-GlcNAc, ThermoFisher, Waltham, MA, USA, Cat#MA1-072, 1:200; WT1, Abcam, Cambridge, UK, Cat#ab89901, 1:200; nephrin, Abcam, Cambridge, UK, Cat#ab216341, 1:200) diluted in blocking solution overnight at 4 °C. After washing with PBS, the slices were incubated with the secondary antibodies for 1 h. Then, the slices were counterstained with DAPI and mounted with an aqueous mounting medium (Sigma, St. Louis, MO, USA).

### 4.5. Immunohistochemistry Staining

The FFPE sections were deparaffinized and rehydrated as previously described [44]. Antigen retrieval was performed by autoclaving at 120 °C for 15 min in citrate buffer, followed by a 1% H_2_O_2_ treatment for 10 min, and 0.2% Triton X-100 was used to permeabilize the membranes. The slices were incubated with blocking solution containing 10% donkey serum for 1 h, and then they were combined with the primary antibodies (phosphor-eIF2α, ProteinTech, Rosemont, IL, USA, Cat#28740-1-AP, 1:2000; ATF4, ProteinTech, Rosemont, IL, USA, Cat#10835-1-AP, 1:2000; ATF6, ProteinTech, Rosemont, IL, USA, Cat#24169-1-AP, 1:2000; WT1, Abcam, Cambridge, UK, Cat#ab89901, 1:2000) and diluted in blocking solution overnight at 4 °C. The slices were incubated with biotinylated secondary antibodies for 1 h, followed by 1 h of incubation with a StreptAvidin–BiotinComplex solution (SABC-POD Kit, Boster Biological Technology Co., Ltd., Wuhan, China). DAB was applied to visualize the antibody labeling and hematoxylin was applied to the slices for the counterstaining.

### 4.6. Immunoblotting

Immunoblotting was performed as previously described [43,44,45]. Briefly, the tissues/cells were homogenized and lysed in a lysis buffer containing protease and phosphatase inhibitors. The same amounts of protein were electrophoresed by sodium dodecyl sulfate–polyacrylamide (SDS-PAGE) gel and then transferred to PVDF membranes (Millipore-Sigma, St Louis, MO, USA). The membrane blotting was performed using 5% nonfat milk for 1 h at room temperature, and then the membranes were incubated with the primary antibodies overnight at 4 ℃. After washing with TBST, the membranes were incubated with HRP-conjugated goat anti-rabbit or goat anti-mouse secondary antibodies for 1 h. The signals were captured using a SuperSignal West Femto Maximum Sensitivity Substrate kit (Thermo Scientific, Rockford, IL, USA).

The following antibodies were used in this study: Xbp1 (ProteinTech, Rosemont, IL, USA, Cat# 24868-1-AP, 1:1000); PERK (ProteinTech, Rosemont, IL, USA, Cat#24390-1-AP, 1:1000); phosphor-PERK (ThermoFisher, Waltham, MA, USA, Cat#PA5-102853, 1:1000); CHOP (ProteinTech, Rosemont, IL, USA, Cat#15204-1-AP, 1:1000); Bip (ProteinTech, Rosemont, IL, USA, Cat#11587-1-AP, 1:1000); Ngly1 (Abclonal, Cambridge, MA, USA, Cat#A22761, 1:1000); O-GlcNAc (ThermoFisher, Waltham, MA, USA, Cat#MA1-072, 1:1000); OGT (Abclonal, Cambridge, MA, USA, Cat#A3501, 1:1000); OGA (ProteinTech, Rosemont, IL, USA, Cat#14711-1-AP); nephrin (Abcam, Cambridge, MA, USA, Cat#ab216341, 1:1000); synaptopodin (Santa Cruz Biothechnology, Santa Cruz, CA, USA, Cat#SC515842, 1:1000); GFAT1 (ProteinTech, Rosemont, IL, USA, Cat#14132-1-AP); GNPNAT1 (ProteinTech, Rosemont, IL, USA, Cat#16282-1-AP); actin (ProteinTech, Rosemont, IL, USA, Cat# 20536-1-AP; 1:10,000); actin (ProteinTech, Rosemont, IL, USA, Cat#66009-1-Ig); goat anti-rabbit secondary antibody (Abcam, Cambridge, MA, USA, Cat#ab205718); and goat anti-mouse secondary antibody (Abcam, Cambridge, MA, USA, Cat#ab205719).

### 4.7. Cell Culture and Treatments

The conditionally immortalized human podocyte (APC) cell line was a kind gift from Dr. Saleem (Children’s Renal Unit and Academic Renal Unit, University of Bristol, UK) [46]. The podocytes were cultured at 33 °C in RPMI 1640 supplemented with 10% fetal bovine serum (FBS), 20 U/mL of mouse recombinant interferon-g (IFN-γ), and 100 U/mL of penicillin plus 0.1 mg/mL of streptomycin for proliferation. For differentiation, the podocytes were maintained at 37 °C without IFN-γ for 7 days, and then they were used for further investigations. To mimic the diabetes condition, the differentiated APC cells were cultured in a high-glucose (30 mM) 1640 medium for 72 h, and the APCs in the control group were seeded in normal glucose (5.5 mM). In different groups, the cells were treated with the 1640 medium supplemented with OSMI1 (50 μM) or Thiamet G (50 μM).

### 4.8. Flow Cytometry Analysis

The cells were harvested and resuspended in FACS buffer (1% FBS plus 0.1% EDTA in Hank’s buffer). For the podocyte pull-down efficiency confirmation, the cells were then incubated with the CD151 antibody (Cat#FAB4609A, R&D System, Minneapolis, MN, USA) for half an hour at 4 °C and protected from light. For apoptosis detection, the assay was conducted according to the manufacturer’s instructions. Briefly, the cells were stained with annexin V-FITC (Cat#640914, Biolegend, San Diego, CA, USA) for 20 min at 37 °C in annexin V binding buffer. Then, propidium iodide (PI) was added to the cells 10 min prior to the running tubes. The signals were recorded using Caton II or LSR Fortessa flow cytometers (BD Biosciences, Franklin Lakes, NJ, USA).

### 4.9. Nephroseq V5 Data Acquisition

Available to the public, Nephroseq V5 (http://v5.nephroseq.org, accessed on 16 December 2022) is a powerful database that integrates many kidney gene expression profiles. It is widely used in data mining and the visualization of gene expression data. The Nephroseq V5 platform was applied to analyze the protein O-GlcNAcylation-related gene expression levels of the healthy kidney donors and the DN patients.

### 4.10. Transmission Electron Microscopy

The tissue samples were fixed with a solution containing 2.5% glutaraldehyde and 2% paraformaldehyde at 4 °C overnight. Then, the samples were washed with 0.1 M sodium cacodylate buffer, treated with 0.1% tannic acid, and post-fixed with 1% osmium and stained en bloc with 0.1% uranyl acetate. The samples were then dehydrated with increasing concentrations of ethanol. After dehydration, the samples were embedded in LX-112 medium, and the ultra-thin sections were cut and post-stained with 2% saturated uranyl acetate and lead citrate. The stained sections were examined using transmission electron microscopy.

### 4.11. Magnetic Microbeads Podocyte Pull-down Assay

The magnetic microbead live-cell pull-down assay was conducted according to the manufacturer’s instructions. Briefly, the cells were resuspended with MACS buffer (1% FBS plus 2Mm EDTA in PBS) and incubated with biotin-conjugated CD151 (Cat#130-109-004, Miltenyi Biotec, Bergisch Gladbach, Germany) antibody for 15 min at 4 °C and protected from light, followed by anti-biotin magnetic microbead (Cat#130-090-485, Miltenyi Biotec, Bergisch Gladbach, Germany) incubation for 10 min under the same condition. The live cells and magnetic microbead mix were added to a column placed in a magnetic multi-stand for further separation. The CD151 cells were harvested after being flushed out of the column.

### 4.12. Plasma Analysis

Plasma urea nitrogen levels were detected using a Urea Nitrogen Colorimetric Detection kit (Cat#C013-2-1, Nanjing Jiancheng Bioengeering Institute, Nanjing, China) according to the manufacturer’s protocol. The plasma creatinine was measured using a Creatinine (Cr) Assay kit (sarcosine oxidase) (Cat#C011-2-1, Nanjing Jiancheng Bioengineering Institute, Nanjing, China) according to the manufacturer’s protocol.

### 4.13. Statistical Analysis

All quantitative data are presented as means ± standard errors (SEs). Student’s *t*-tests were applied to the two-group comparisons. One-way and two-way ANOVA tests were used for comparisons that included more than two groups, and Tukey tests were used to estimate the significance between groups once significant differences were found. Differences were considered statistically significant when *p* < 0.05. All statistical analyses were performed using the GraphPad Prism V8 software.

## 5. Conclusions

The conducted study found that hyperglycemia-induced ER stress occurred prior to protein O-GlcNAcylation, and thus, it initiated podocyte apoptosis. Both an ER stress inhibitor and an O-GlcNAc modification inhibitor could alleviate podocyte injuries, which could help deepen our understanding of the mechanisms mediating podocyte injury under a DN condition and lead to novel therapeutics. Based on the findings of the present study, reducing modifiable ER stress and O-GlcNAcylation exposures may be an effective approach for improving clinical outcomes in DN.

## Figures and Tables

**Table 1:** ER stress-perturbed intracellular protein O-GlcNAcylation aggravates podocyte injuries in diabetes nephropathy.

	ND	db/m	STZ plus UNx	db/db plus UNx
Body weight, baseline (g)	24.4 ± 2.71	23.67 ± 3.66	24.8 ± 3.21	29.5 ± 3.72
Body weight, endpoint (g)	34.7 ± 2.44	36.72 ± 4.23	25.9 ± 2.62 *	42.3 ± 3.87 #
Glucose, baseline (mmol/L)	5.3 ± 0.65	6.11 ± 0.35	4.7 ± 0.67	14.3 ± 3.41
Glucose, endpoint (mmol/L)	4.9 ± 0.52	5.54 ± 0.46	26.7 ± 4.46 *	28.3 ± 4.84 #
Plasma creatinine (mg/dL)	0.22 ± 0.07	0.16 ± 0.09	0.53 ± 0.06 *	0.62 ± 0.10 #
Plasma BUN (mg/dL)	19.62 ± 4.66	17.34 ± 5.72	28.55 ± 6.86 *	35.69 ± 5.12 #
Urinary albumin excretion (mg/d)	8.67 ± 2.88	9.32 ± 3.31	233.72 ± 65.43 *	271.26 ± 73.41 #

* and # indicate *p* < 0.05 compared with the ND or db/m groups using Chi-square tests, respectively. ND, non-diabetes and UNx, unilateral nephrectomy.

## Data Availability

All data supporting the findings of this study are available within the article and its supplementary files or from the corresponding author on reasonable request.

## Supplementary Materials

The supporting information can be downloaded at https://www.mdpi.com/article/10.3390/ijms242417603/s1.
