# Supplementary material for: ER Stress-Perturbed Intracellular Protein O-GlcNAcylation Aggravates Podocyte Injury in Diabetes Nephropathy
Source: Int J Mol Sci. 2023 Dec 18;24(24):17603. doi: 10.3390/ijms242417603 (PMC10743520; doi:10.3390/ijms242417603)
Supplement: Supplementary file 1 [file ijms-24-17603-s001.zip › ijms-2717023-supplementary.pdf]

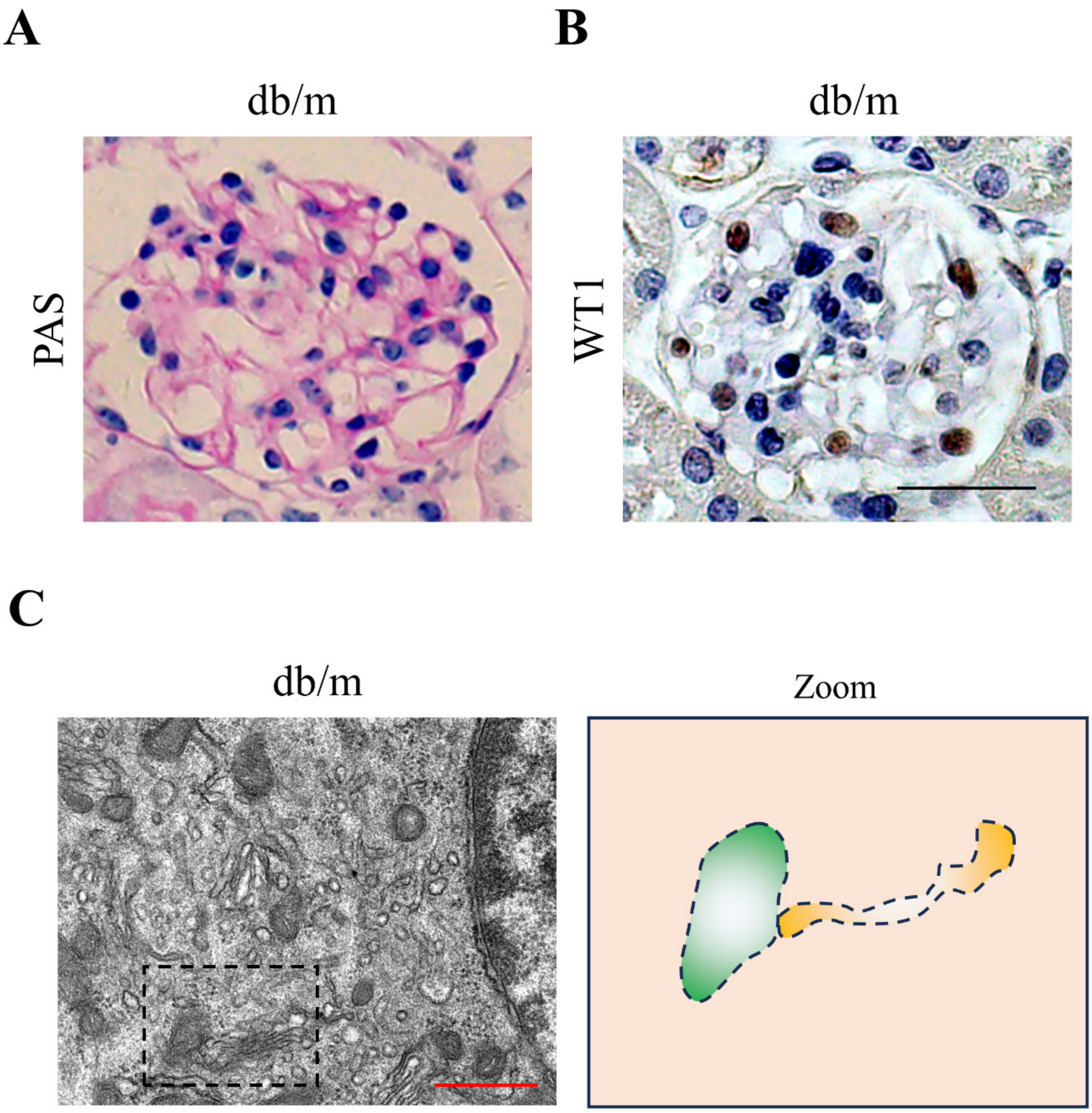

Figure S1: Normal glomerular structure, podocyte number, and subcellular structure in db/m group. (**A**) Glomerular structure of db/m mouse was assessed by periodic acid–Schiff (PAS) staining. (**B**) Representative immunohistochemistry images of WT1 in the kidneys from db/m mouse. (**C**) Representative TEM images of podocytes in the kidneys from db/m mouse, squares highlight the magnified areas and tracing of the mitochondria and ER from the aforementioned TEM images were shown in right panel. Scale bars, 20 µm in (**A, B)**; 0.5 µm in (**C**).

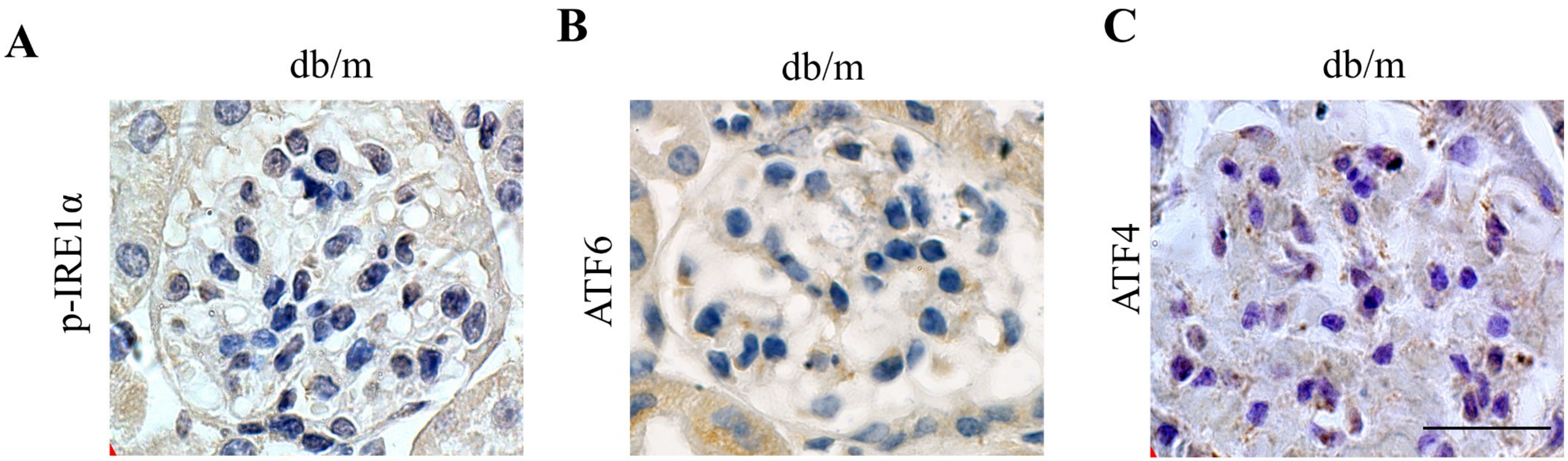

Figure S2: ER stress of db/m was evaluated by immunohistochemistry. Representative immunohistochemistry images of p-IRE1α (**A**), ATF6 (**B**), ATF4 (**C**) in the kidneys from db/m mouse. Scale bars, 20 µm.

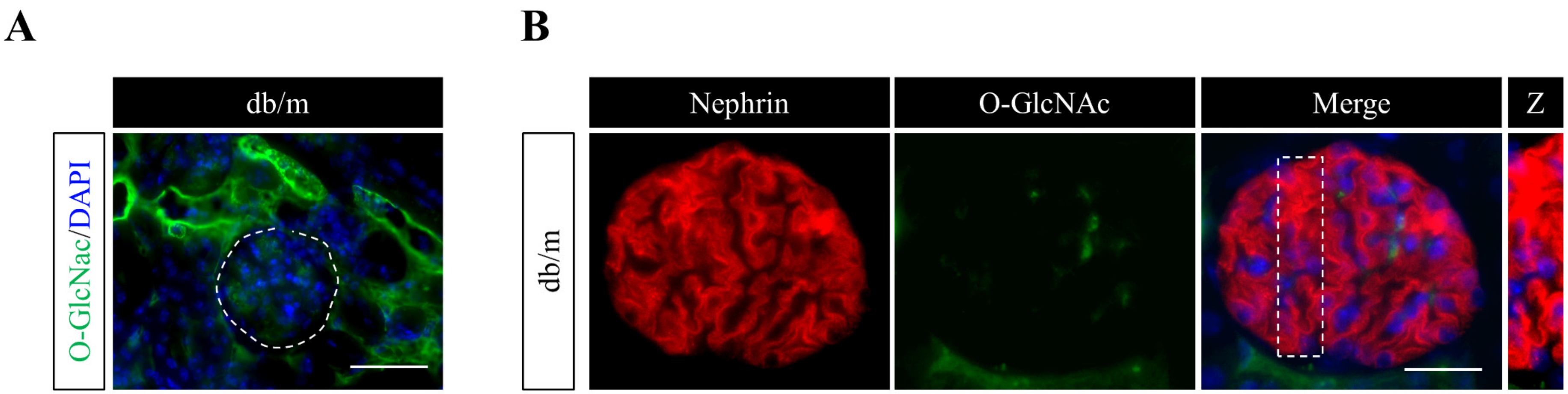

Figure S3: O-GlcNAc protein modification in db/m mouse. (**A**) Representative immunofluorescence images of O-GlcNAc staining in the kidneys from ddb/m, dash circle highlight the glomerulus. (**B**) Representative dual-immunofluorescence staining image of db/m mouse, squares highlight the representative colocalization areas. Scale bars, 10 µm in (**A**) and 20 µm in (**B**).
